# Regulation of WDFY1 Expression by miRNAs, Transcription Factors, and IL-6 in Murine Mesangial Cells

**DOI:** 10.3390/cells14110798

**Published:** 2025-05-29

**Authors:** David E. Adams, Siru Li, Yuxuan Zhen, Ahmet Kaynak, Xiaoyang Qi, Jane J. Yu, Wen-Hai Shao

**Affiliations:** 1 Division of Rheumatology, Allergy & Immunology, Department of Internal Medicine, College of Medicine, University of Cincinnati, Cincinnati, OH 45267, USA; david.adams@cchmc.org (D.E.A.);; 2 Division of Immunobiology, Cincinnati Children’s Hospital Medical Center, Cincinnati, OH 45267, USA; 3 Division of Pulmonary Disease, Department of Internal Medicine, College of Medicine, University of Cincinnati, Cincinnati, OH 45267, USA; 4 Division of Rheumatology, Cincinnati Children’s Hospital Medical Center, Cincinnati, OH 45267, USA; 5 Division of Hematology & Oncology, Department of Internal Medicine, College of Medicine, University of Cincinnati, Cincinnati, OH 45267, USA; kaynakat@ucmail.uc.edu (A.K.); qix@ucmail.uc.edu (X.Q.)

**Keywords:** WDFY1, transcription factor, miRNA, luciferase, IL-6

## Abstract

WD40 repeat and FYVE containing protein 1 (WDFY1) functions in membrane trafficking and protein complex scaffolding. WDFY1 has been studied in the immune system and in different oncogenic conditions. Therefore, comprehensive understanding of WDFY1 regulation mechanisms is much desired. In this study, we analyzed the promoter and 5′- and 3′-untranslated regions (UTRs) of *wdfy1* and identified critical sequence elements, transcription factors (TFs), and miRNAs that collaboratively regulate *wdfy1* gene expression. A 3.5 kb segment of the mouse *wdfy1* promoter and 5′-UTR was cloned into a luciferase expression vector and transfected into HeLa cells. Luciferase assays of promoter deletion mutants revealed approximately four-fold increased activity attributed by a 500 bp distal fragment upstream of the *wdfy1* 5′-UTR. Four TFs (Sp1, Ap-1, Hes1, and TCF7) were found to be critical for *wdfy1* expression with binding sites spread throughout the promoter and 5′-UTR regions. Cloning of a 3.2 kb fragment of *wdfy1* 3′-UTR into the luciferase expression vector led to an ~3.5-fold decrease in luciferase activity. Complementary siRNA and luciferase assays mutually confirmed our findings. Most importantly, IL-6, a critical cytokine in organ inflammation, was found to promote WDFY1 expression through the upregulation of Sp1 in primary renal mesangial cells. We, therefore, identified a potential inflammation-driven WDFY1 upregulation in mice.

## 1. Introduction

WD40 repeat and FYVE containing protein 1 (WDFY1) contains seven WD40 repeats and a FYVE zinc finger domain [1]. The FYVE domain is required for high-affinity binding to phosphatidylinositol 3-phosphate (PI3P) [2], which is important for WDFY1’s localization to early endosomes [3,4]. WD40 repeats mediate vesicle trafficking and is essential for the interaction with other proteins [5]. WDFY1 acts as an adaptor protein for the TLR3/4 signaling pathways and serves to mediate innate and adaptative immune responses by recruiting TIR domain-containing adapter-inducing interferon-β (TRIF) to the proximal endosomal membrane [6,7]. However, WDFY1 mainly participates in TLR3-mediated innate immune responses but not TLR4-mediated host defense [8], since TLR4 can also signal through the MyD88-dependent pathway [9]. WDFY1 is also localized to lysosomes, being part of an E3 ubiquitin ligase complex involved in selective autophagy recognizing and removing damaged lysosomes [10].

The *Mus musculus* WDFY1 gene is located on chromosome 1qC4. It is highly conserved, and 233 organisms have orthologs with humans. Differential *wdfy1* expression has been reported by different groups via mega data analysis in gene expression and regulation studies [11]. *Wdfy1* is aberrantly upregulated in the hippocampus and striatum of mice devoid of transcription factor (TF) Helios [12]. Consistent with these observations, differential WDFY1 expressions have been reported to be associated with schizophrenia [13]. Aberrant WDFY1 expression has also been associated with mitochondrial dysfunction related to Alzheimer’s disease [14]. WDFY1 is highly expressed in mouse testes and locates in the cytoplasm of late pachytene spermatocytes to elongated spermatids, but its function is dispensable for mouse spermatogenesis [11]. We have previously reported a critical function for WDFY1 in follicular dendritic cell (FDC) antigen presentation. Reduced autoimmune response was noticed in WDFY1-deficient mice compared with WDFY1-sufficient mice [15]. Therefore, it is increasingly critical to understand the mechanisms that regulate *wdfy1* gene transcription and translational regulation. In this study, we delineated several mechanisms that contributed to the base level expression, regulation, and inflammatory stimulation of *wdfy1.* The *Wdfy1 3*′ and 5′ untranslated regions (UTRs) and ~3.0 kb of upstream sequences were cloned into a luciferase reporter vector. Sequential 5′ deletion and miRNA target site deletion of selected sequence elements helped uncover critical TFs and miRNAs important for *wdfy1* expression. The miRNA knockdown results were validated by complementary miRNA mimic experiments. Most importantly, we discovered an IL-6-regulated induction of WDFY1 expression through the induction of Sp1 expression.

## 2. Materials and Methods

### 2.1. Reagents

*mir*Vana miRNA mimics (hsa-miR-29b-3p, Assay ID MC10103; mmu-miR-124-3p, Assay ID MH10691) were obtained from Life Technologies Corporation (Carlsbad, CA, USA). All enzymes used in cloning were from either New England BioLabs (Ipswich, MA, USA) or ThermoFisher Scientific (Waltham, MA, USA). Anti-WDFY1 Ab (1:500) was purchased from Aviva Systems Biology (San Diego, CA, USA). All other Abs including anti-GAPDH and anti-Sp1 were purchased from Cell Signaling Technology (Danvers, MA, USA). All Silencer Select siRNAs were premade at ThermoFisher.

### 2.2. PCR Cloning of Wdfy1 Promoter and 5′ UTR

A 7.0 kb region encompassing the *Mus musculus* C57BL/6 *wdfy1* promoter and 5′ UTR regions was downloaded from NCBI Genbank and analyzed using PROMO (version 3.0) and pDRAW32 (version 1.1.151), and Clustal Omega (EMBL-EBI) online programs. PCR primers were designed to amplify the 3.5 kb of the proximal promoter and 5′ UTR regions, which included the main transcriptional starts sites. The primers were designed with the aid of Primer 3, a Primer-BLAST tool (NIH National Library of Medicine), after inputting 3500 bp of the *wdfy1* proximal promoter/5′ UTR sequence derived from C57BL/6 mice. Seven PCR primers with different combinations were designed: F3 (5′ GGACT CCCTC GAGGC TACCA TC 3′), F4 (5′ CCAGT AGCTC GAGTT CTAAC TCTG 3′), R3 (5′ CCGCC GCCAT GGTCG AG 3′), R4 (5′ GCCGC CATGG TCGAG CC 3′), F1 (5′ GGCCT AACTG GCCGG GCTTG GTAGT GGA 3′), F2 (5′ GGCCT AACTG GCCAG GTCCT TGACC TTTTC ACAT 3′), and R2 (5′ GGCCG CCGAG GCGCG GGCGG CCCAT CAG 3′). The *wdfy1* promoter and 5′-UTR regions were amplified with the high-fidelity DNA polymerase (Takara Bio, San Jose, CA, USA). The primer pair F3–R4 generated a 3.5 kb PCR product and the primer pair F4–R4 generated a 1.9 kb PCR product, whereas the primer pair F1–R2 generated a 3.4 kb PCR product, and the primer pair F2–R2 generated a 2.4 kb PCR product. To prepare these products for DNA ligation, the pGL4.0 luciferase vector (KA1390, Addgene, Watertown, MA, USA) and PCR products were restricted with either *Xho*I or *Nco*I (F3–R4 and F4–R4 products) or *Bgl*I (F1–R2 and F2–R2 products).

### 2.3. PCR Cloning of Wdfy1 3′ UTR

A 3.5 region encompassing the C57BL/6 mouse *wdfy1* 3′ UTR was downloaded from NCBI Genbank and analyzed using the TargetScanMouse (Release 8.0) and pDRAW32 (version 1.1.151) online programs. Primers were designed to amplify up 3.2 kb of this region, starting from +5 bp downstream from the *wdfy1* stop codon and extending to +3143 bp downstream. Within the amplified sequence, there is a B1 SINE element that lies at +1351 to +1487. The primers used for PCR amplification are F5 (5′ CCTCT AGAGC TGGGT GATGT CTGCT A 3′) and R5 (5′ CGCGG CCGGC CGGAA CAAGC AAGGG GTATT TTAT 3′) that generated a 3.2 kb product. The recovered PCR fragments were digested with restriction enzymes *Xba*I and *Fse*I to prepare the DNAs for ligation to pGL4-Wdfy1-1.4-luc2. Following ligation and transformation into *E. coli Stbl3* cells, resistant colonies were selected by using LBcarb100 plates, and candidate products were miniprepped and screened to obtain the pGL4-1.4-luc2-3.2 construct.

### 2.4. Generation of Luciferase Reporter Constructs

The full-length *wdfy*1 promoter/5′-UTR and 3′-UTR was sequence verified at CD Genomics (New York, NY, USA). The sequential deletions of the *wdfy1* promoter were also generated using time-restricted exonuclease III digestion. Blunted resected ends were then ligated by incubating with T4 DNA ligase at 16 °C overnight. Recombinant clones were isolated after transformation of the ligation products into *E. coli Stbl3* cells (Invitrogen, Waltham, MA, USA) and selection of drug-resistant colonies on LBcarb100 plates. Each DNA construct was size- and sequence-verified by agarose gel electrophoresis and nanopore sequencing (CD Genomics), respectively.

### 2.5. Luciferase Assay

HeLa cells (ATCC, Manassas, VA, USA) were cultured in Gibco DMEM medium (10% FBS and 2 mM L-glutamine). Twenty-four hours before the experiment, cells were seeded into 24-well plates at 7.5 × 10^5^ cells per well. Full-length pGL4-Wdfy1 3.5-luc2 DNA or vectors with various lengths of the promoter (128 ng) were diluted into Opti-MEM medium containing Lipofectamine 3000 and P3000 reagent (Invitrogen, Waltham, MA, USA) and either transfected alone or co-transfected with 125 ng of TF expressing vector (GeneCopoeia, Rockville, MD, USA). The cells were lysed with the Steady-Glo luciferase reagent (Promega, Madison, WI, USA) after 24 h post-transfection culture. Luminescence was measured with the EnVision plate reader (PerkinElmer, Waltham, MA, USA) within 60 min.

### 2.6. Manipulation of Wdfy1 Expression Using miRNA Mimics and siRNAs in HeLa Cells

*Mir*Vana miRNA mimics (hsa-miR-29b-3p and mmu-miR-124-3p) that targeted the *wdfy1* 3′-UTR were purchased from Life Technologies Corporation (Carlsbad, CA, USA). Ten nM of each mimic was added into HeLa cell cultures transfected either with the pGL4-1.4-luc2-3.2 construct or with one of two different microRNA target site deletion constructs, pGL4-1.4-luc2-3.2-Δ*miR*-*29*-*3p* or pGL4-1.4-luc2-3.2-Δ*miR*-*124*-*3p*. Luciferase expression was measured 24 h post-transfection.

### 2.7. Mesangial Cell Culture, siRNA Treatment, and IL-6 Stimulation

Mesangial cells (ATCC) were cultured in ATCC formulated DMEM medium and Ham’s F12 medium at 3:1 ratio, with 14 mM HEPES and 5% FBS. For siRNA treatment, cells were sub-cultured into 6-well plates at a density of 2 × 10^5^ cells per well. The next day, siRNAs (30, 60, or 90 pM) targeting the specific gene were mixed with 9 μL of lipofectamine (Thermofisher) for 15 min and then added into the culture. Two days later, the mesangial cells were washed twice with cold 1 × PBS and lysed in RIPA buffer (Santa Cruz Biotechnology, Dallas, TX, USA) and incubated at 4 °C for 1 h. After centrifugation at 13,000 rpm for 10 min, supernatant (cell lysate preps) was stored at −80 °C for Western analysis. For IL-6 treatment, primary mesangial cells (ScienCell, Carlsbad, CA, USA) were cultured in 6-well plates (0.5 × 10^6^ cells/well) and then activated with the recombinant mouse IL-6 (rmIL-6, 0.1, 1, 10, and 100 ng/mL, Sigma-Aldrich, St. Louis, MO, USA) for 24 h. Cell lysates were prepared in RIPA buffer and processed for Western analysis.

### 2.8. Western Blot Analysis

Cell lysate preps were mixed with 4× sample loading buffer plus reducing reagent and loaded onto pre-cast NuPAGE Bis-Tris Gels (Invitrogen, Carlsbad, CA, USA) and separated using 100 volts for 1.5 h. Protein bands were then transferred onto the PVDF membrane. For immunoblotting, membranes were blocked with 5% non-fat dry milk in TBST buffer (Tris-based buffer with 0.1% Tween-20) for 1 h at room temperature (RT), then incubated with primary antibodies in fresh blocking buffer for 1 h at RT. After washing 3 times with TBST buffer, membranes were incubated with the cognate HRP-conjugated secondary antibodies. ECL buffer was then added to the membranes after washing. Film was developed with various times of exposure.

### 2.9. Statistical Analysis

GraphPad Prism 10 (San Diego, CA, USA) was used to perform all statistical analyses. After Welch’s correction, a Student’s *t*-test was applied to analyze the luciferase activities between two groups. One-way ANOVA with a post hoc test was applied to multi-group tests. Western protein band intensity was analyzed using the Image Studio software (v4.0.21). The Mann–Whitney U test was applied to analyze the differences between groups. Data are shown as the median with interquartile range.

## 3. Results

### 3.1. Cloning and Identification of Putative Regulatory Elements Within the Wdfy1 Promoter and 5′-UTR Regions

**Figure 1:**
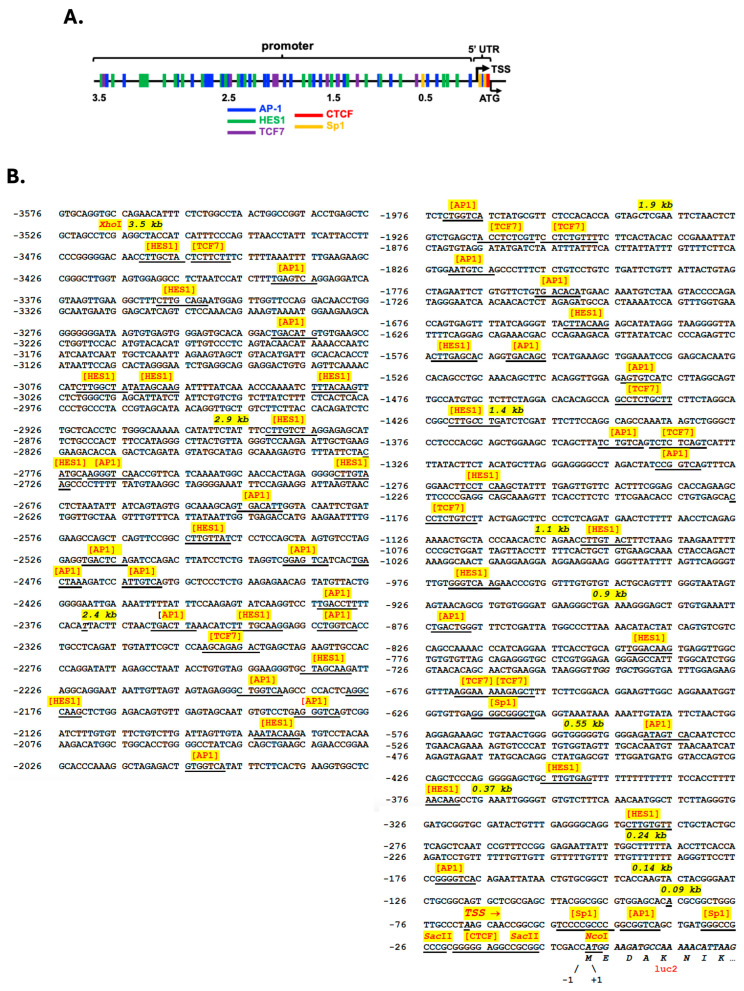
Analysis and cloning strategies of *wdfy*1 5′-UTR. (**A**) Characterized potential TF binding sites at the *wdfy1* 5′-UTR and promoter regions. Five TF binding sites were cross-identified by two independent online softwares with 15% dissimilarity as indicated in the methods. TF biding sites are color coated in the schematic line. (**B**) Schematic representation of the 5′ flanking region of *wdfy1* promoter with putative TF binding sites indicated in A. Restriction enzyme binding sites for cloning are also marked. The sizes and sites of serial deletions are also marked. TSS, transcription start site.

WDFY1 expression has been associated with many diseases, including lupus [15]. In this study, we identified candidate TF binding sites in the promoter and 5′ UTR regions of *wdfy1* by uploading the sequence onto the online software, PROMO and pDRAW32, respectively. Five prominent TFs (AP-1, Sp1, HES1, TCF7, and CTCF) were selected for further investigation based on the maximum 15% dissimilarity (Figure 1A). There were a total of 23 predicted HES1 and AP-1 binding sites and 7 TCF7 binding sites (Figure 1B). These were scattered across the whole length of the 3.5 kb promoter region. However, there were only three Sp1 binding sites and one candidate CTCF binding site. All three Sp1 binding sites were proximal (within 600 bp range) to the ATG start codon (Figure 1B), whereas the CTCF binding site was about 15 bp from the start codon (Figure 1B).

**Figure 2:**
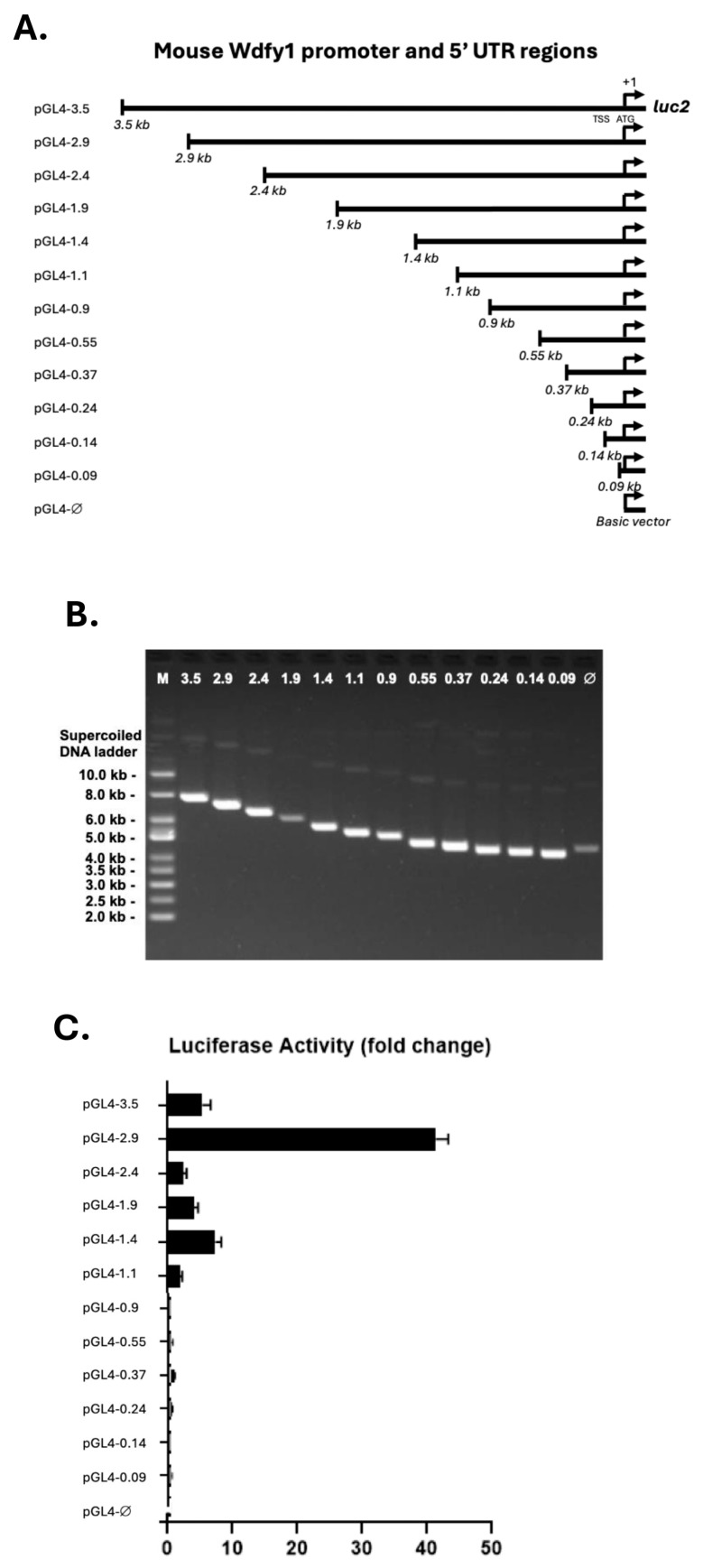
Construction of serial deletions of the *wdfy1* promoter and luciferase activity assays. (**A**) Constructs of the sequential deletions of the WDFY1 promoter region. (**B**) Representative image of WDFY1 serial deletion constructs. (**C**) HeLa cell luciferase activities were assayed 24 h after transit expression of the correspondent luciferase reporter constructs (pGL4-0.09 to pGL4-3.5). The fold change was calculated by dividing the serial deletion construct luciferase activities by the basal pGL4-Ø luciferase activity. The data are representative of the two repeats.

To define the limits of the *wdfy1* promoter and identify potential cis-regulatory elements involved in its transcriptional regulation, we isolated the 5′ flanking region of 3.5 kb upstream of the translation start site (TSS). The sequence did not contain typical TATA and CAAT boxes (Figure 1B). Based on potential cis-regulatory elements and major transcriptional start sites, we generated a series of 5′ promoter deletion constructs (500 bp apart, total 12 constructs) as outlined in Figure 2A and Table S1 and size verified in Figure 2B. The human HeLa cell line lacking mouse TF factors was used for the luciferase reporter study. To determine the minimal *wdfy1* promoter region and to narrow down cis elements regulating basal *wdfy1* gene expression, we transfected a series of promoter deletion constructs (Figure 2C) into HeLa cells and then measured the luciferase activity. The construct pGL4-2.9 (−2.9 kb) exhibited the maximum fold increase in luciferase activity as compared with the other constructs (Figure 2C). The full-length construct and all other deletion derivatives showed significantly less (about four times decreased) luciferase activity in HeLa cells. Further analysis did not reveal any additional regulatory elements (either inhibitory or enhancement) between −3.5 and −2.4 kb upstream from the reporter ATG (Figure 1B). However, this region contained nine HES1, eight AP-1, and one TCF7 predicted binding site, the densest concentration of TF sites in the area, indicating a possible distal add-up effect on the TF promoted *wdfy1* transcription. The last 1.0 kb of the promoter region did not contribute to basal *wdfy1* transcriptional activity. A minimum 1.1 kb of the promoter region was required to detect any luciferase activity higher than the non-promoter, control luciferase vector (Figure 2C). Taken together, where the maximum promoter activity lies within the 2.9 kb region, the data suggest that a 1.1 kb fragment upstream of TSSs is required to promote significant *wdfy1* transcription.

### 3.2. Multiple TF Regulated WDFY1 Expression in Mesangial Cells

**Figure 3:**
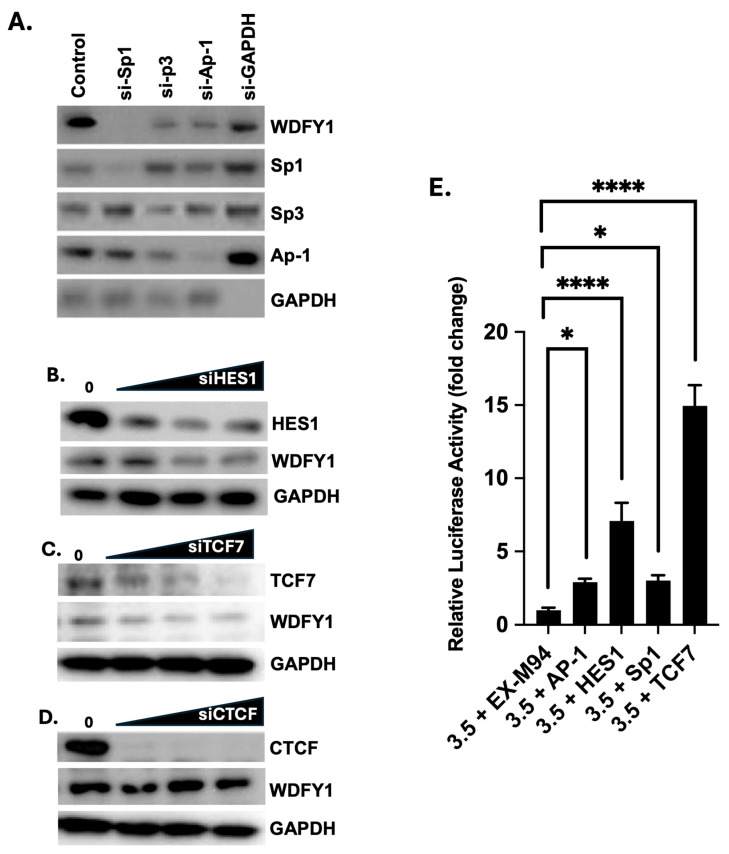
TF regulated WDFY1 expression in mesangial cells. Mesangial cells were treated with siRNAs (Sp1, Sp3, and Ap-1) in (**A**), Hes-1 (**B**), TCF7 (**C**), and CTCF (**D**). WDFY1 and TF expression levels were analyzed by Western blot. GAPDH expression served as an internal siRNA control and protein loading control. The experiment was repeated three times. (**E**) HeLa cells were transiently transfected with luciferase reporters (pGL4-3.5) and TF expressing constructs. Luciferase activity was measured as described in the methods. Experiments were repeated twice. One-way ANOVA with a post hoc test was performed to compare relative luciferase activities of different constructs with the basal full-length pGL-3.5 WDFY1 construct. * *p* < 0.05, **** *p* < 0.0001.

Two experimental approaches were applied to investigate the importance of the previous identified TF binding sites. We first treated mesangial cells with TF individual targeting siRNAs and then measured WDFY1 expression by Western blot. As shown in Figure 3A–D, siRNA-mediated knockdown of TF expression diminished Sp1, Ap-1, Hes1, CTCF, and TCF7. Four TFs, i.e., Sp1, Ap-1, Hes1, and TCF7, were found to directly regulate WDFY1 expression as decreased levels of WDFY1 protein were detected by Western blot when mesangial cells were treated with the cognate TF-targeted siRNAs (Figure 3A–C). However, a fifth TF, CTCF, identified by the same software, when knocked down completely by siRNA in mesangial cells, showed no noticeable alteration of WDFY1 expression (Figure 3D), suggesting that CTCF may not regulate WDFY1 expression in mesangial cells. In addition, we also included Sp3 siRNA-treated mesangial cells in this experiment (Figure 3A) because Sp3 and Sp1 were closely related and recognized and associated with the same GC-rich DNA motifs with similar affinity [16]. Sp3 often serves as a repressor for Sp1 [17,18]. To our surprise, Sp3 knockdown also decreased WDFY1 expression in mesangial cells (Figure 3A). In summary, four prominent TFs (Sp1, Ap-1, Hes1, and TCF7) were identified to regulate WDFY1 expression in renal mesangial cells.

Next, HeLa cells were co-transfected with the full-length vector (pGL4-3.5) and individual TF vectors that were commercially available. The original vector plasmid (EX-M94) without TF expressing genes served as the control. Luciferase activity was recorded and analyzed over the baseline pGL4 empty vector. Figure 3E shows a significantly increased luciferase activity in HeLa cells co-transfected with mouse TFs, TCF7, Sp1, Hes1, or Ap-1, compared with the luciferase activity co-transfected with the control vector. It would be interesting to see if co-transfection with CTCF vector leads to an alteration of luciferase activity compared with the control. However, the CTCF expression vector was not commercially available at the time of this study. In summary, four TFs that contributed to WDFY1 upregulation were identified and confirmed in different cell lines, excluding cell-line-specific biases. However, we cannot exclude any indirect effect of a specific TF. This may involve much complex analysis with the help of a 3-D computational model of possible interactions among TFs and other regulatory elements.

### 3.3. IL-6-Induced WDFY1 Upregulation Is Associated with Decreased Sp1 Expression

**Figure 4:**
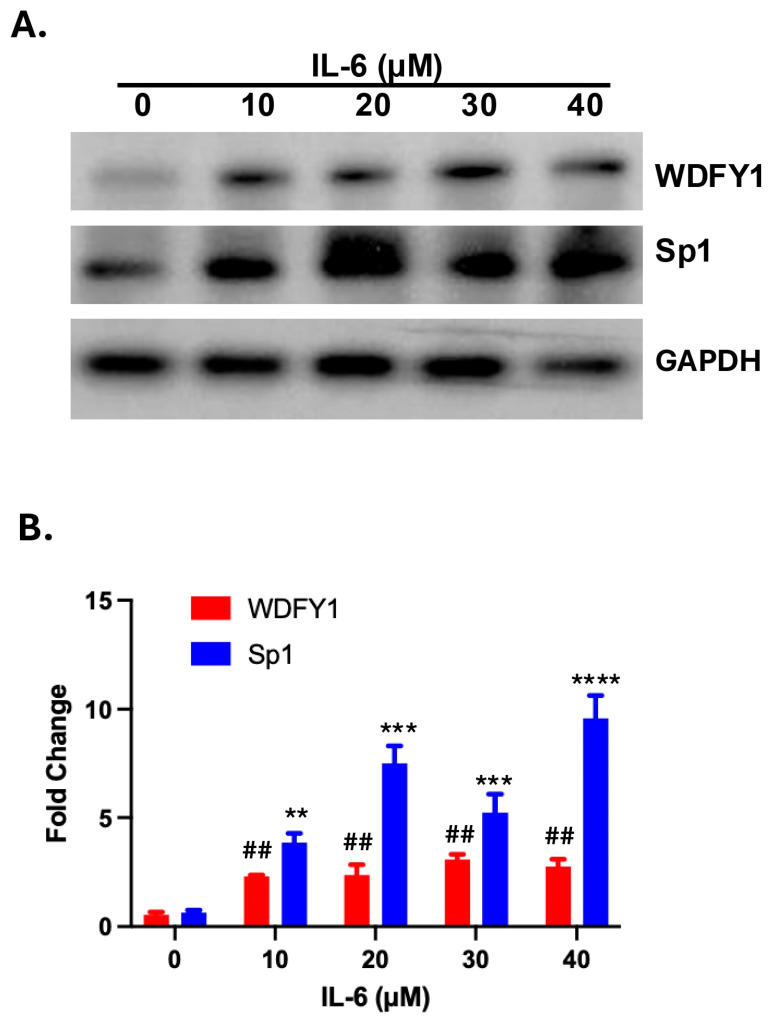
IL-6-induced WDFY1 expression and Sp1 upregulation. (**A**) Primary mesangial cells were treated with increased concentrations of rmIL-6. WDFY1 and Sp1 expressions were analyzed after 24 h. Qualified data are presented in (**B**). Fold changes were calculated against the intensity without rmIL-6 treatment after internal correction with GAPDH. The experiments were repeated twice. Mann–Whitney U test. ** *p* < 0.01, *** *p* < 0.001, **** *p* < 0.0001. Sp1 expression compared with the control; ## *p* < 0.01. DFY1 expression compared with the control.

IL-6 is a pro-inflammatory cytokine that plays a pathological role in several types of nephritis [19]. We observed a greatly increased expression of Sp1 in the kidney of lupus nephritis mice, and the elevated expression levels corelated with the severity of lupus nephritis [20]. Additional studies suggested that IL-6 could induce Sp1 expression in cells [21]. We wondered if upregulation of WDFY1 could be induced by the IL-6-driven upregulation of Sp1 expression in the kidney of lupus nephritis mice. We therefore treated primary renal mesangial cells with increased concentrations of recombinant IL-6 for 48 h and measured protein levels of WDFY1 and SP1 by Western blot. As anticipated, WDFY1 expression was low in primary mesangial cells without inflammatory insult (Figure 4A, top panel). IL-6 significantly enhanced WDFY1 expression in the treated primary renal mesangial cells. This enhancement was associated with increased Sp1 upregulation in the same cells (Figure 4B). We therefore identified an inflammation-induced upregulation of WDFY1 expression in the murine kidney.

### 3.4. 3′-UTR Binding by miRNAs Inhibits WDFY1 Expression

**Figure 5:**
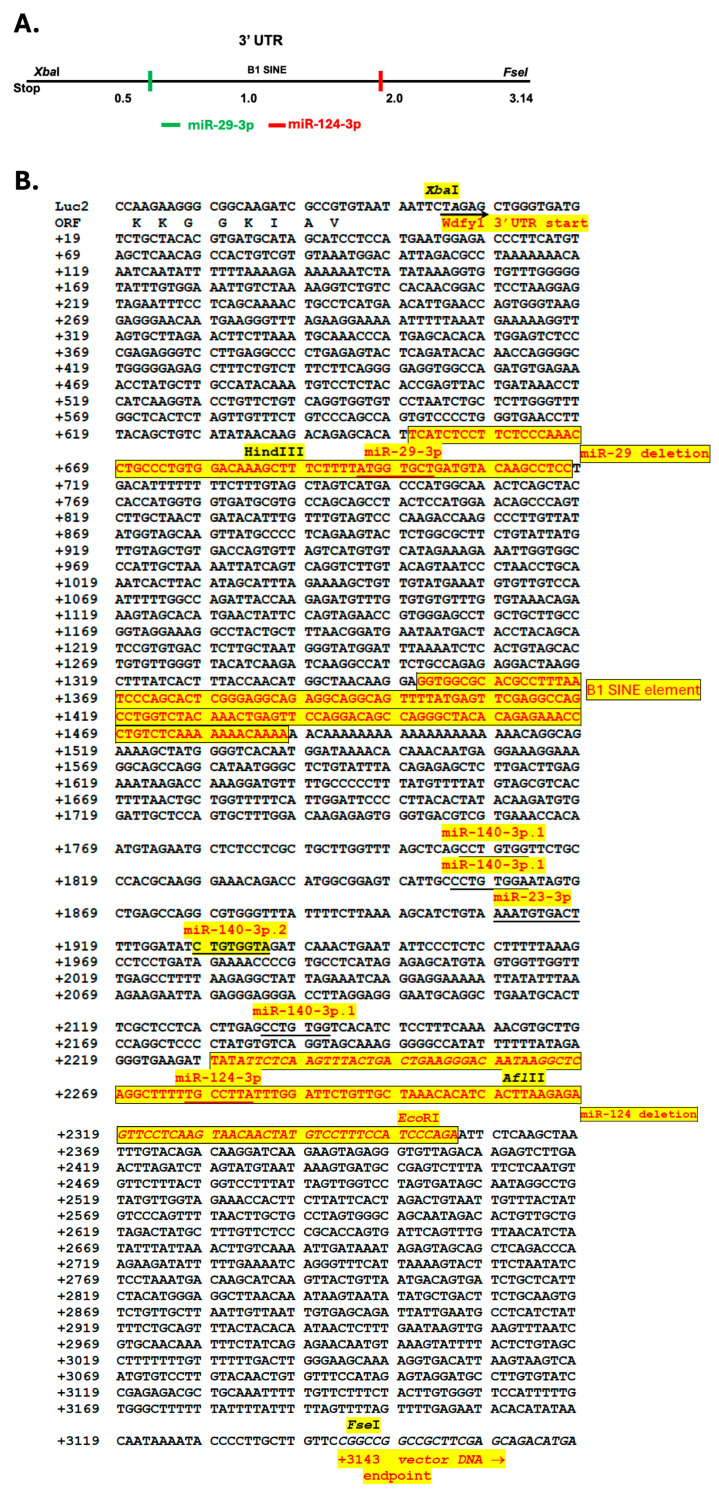
Analysis and cloning strategies of wdfy1 3′-UTR. (**A**) Potential miRNA binding sites are marked. (**B**) Sequences of the 3′ flanking region of *wdfy1* 3′-UTR are shown. The deleted regions that expand the miR-29 and miR-124 binding sties and the B1 SINE element are also highlighted.

**Figure 6:**
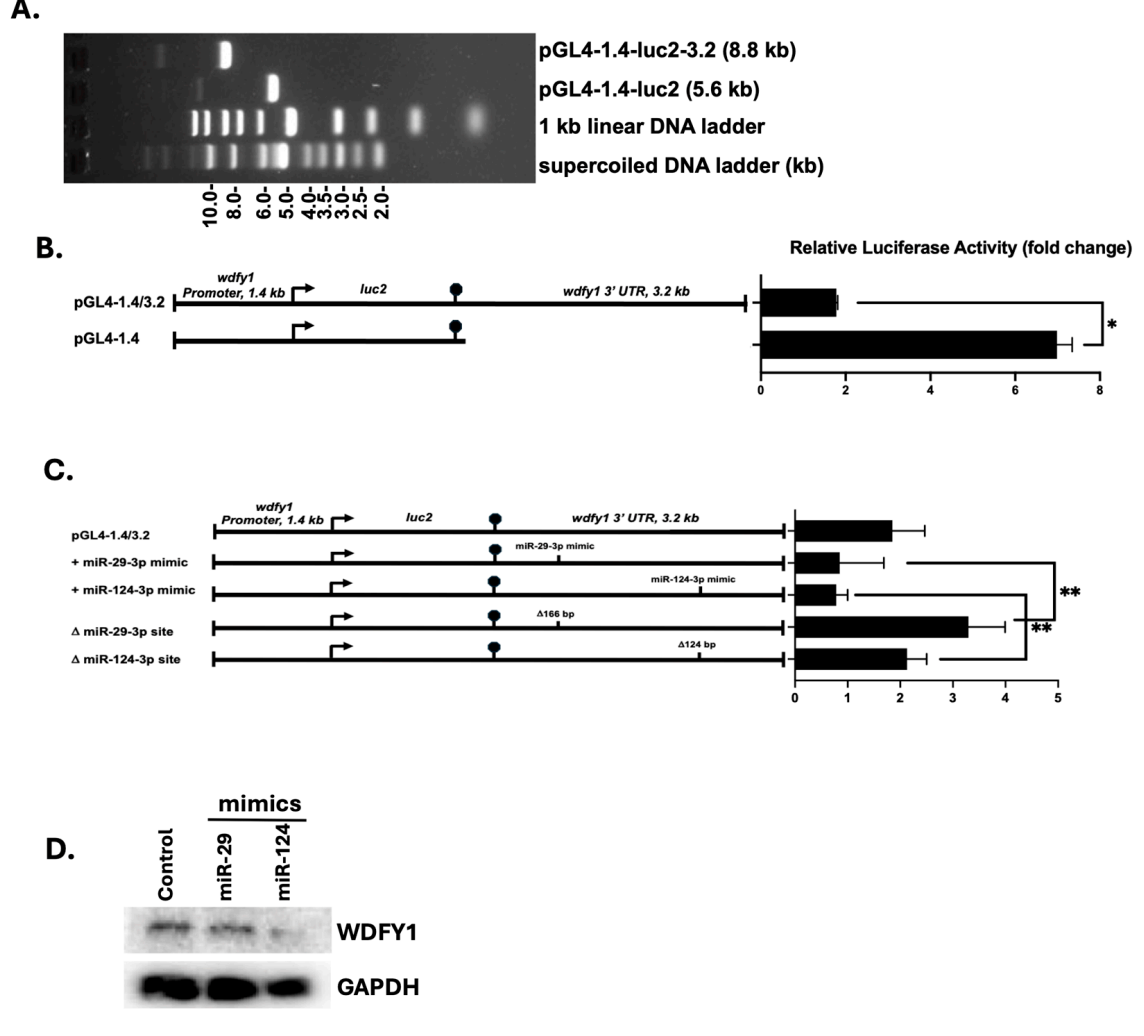
MiR-29 and miR-124-regulated luciferase activities in HeLa cells. (**A**) Gel electrophoretic analysis of the pGL4-1.4 luciferase vector with or without the 3′-UTR 3.2 kb fragment. (**B**) HeLa cells were transiently transfected with the luciferase vectors from (**A**). (**C**) HeLa cells were also transfected with the luciferase vectors plus miR-29 and miR-124 mimics or with deletions of the respective miRNA binding sites. Luciferase activity was assayed 24 h after DNA transfection. The fold change was calculated by dividing the 3′-UTR construct luciferase activities by the basal pGL4-luc vector luciferase activity. The data shown are representatives of the two repeats. Luciferase activities across all constructs were compared using a Student’s *t*-test (**B**) or one-way ANOVA (**C**). * *p* < 0.05, ** *p* < 0.01. (**D**) Mesangial cells were treated with miR-29 or miR-124. WDFY1 expression was detected by Western blot in cell lysate after 48 h. GAPDH served as the internal control.

We analyzed the 3.14 kb fragment of *wdfy1* 3′-UTR and identified a miR-29-3p binding site with high probability of preferential conservation (prediction of microRNA targets at TargetScanMouse 8.0 online software) (Figure 5). A miR-124-3p binding site was also identified but with lower predicted probability. MiR-124 expression was significantly reduced in patients with active LN, and it was negatively correlated with serum inflammatory cytokine expression [22,23]. In addition, miR-124 polymorphism was associated with SLE susceptibility [24]. Consistent with these observations, we found WDFY1 upregulation in the kidneys of SLE patients and mouse lupus nephritis (unpublished data). We therefore included miR-124 in this study (Figure 5). Our analysis of the *wdfy1* 3′-UTR also revealed a B1-Short Interspersed Nuclear Element (SINE) element at +1350 downstream of the *wdfy1* open reading frame (ORF) (Figure 5B). B1-SINEs are a prominent feature of the rodent genome, particularly in mice and rats, and can influence gene expression by interacting with TFs and modulating chromatin structure, potentially acting as insulators or enhancers [25]. Next, we evaluated the role of miR-29-3p and miR124-3p binding sites in WDFY1 expression since both miRNAs were reported to be involved in immune regulation, particularly associated with lupus and lupus nephritis, which was our research focus. We first inserted the *wdfy1* 3′UTR immediately downstream of the luciferase ORF (Figure 6A). We then transfected HeLa cells with the pGL4-1.4 vector with or without the 3.2 kb *wdfy1* 3′-UTR to study miRNA-inhibited reporter expression by measuring the luciferase activity. Insertion of the *wdfy1* 3′-UTR into the pGL4-1.4 vector resulted in a ~3.5-fold decrease in luciferase activity compared with the luciferase activity from the pGL4-1.4 transfected HeLa cells (Figure 6B). Next, we selectively deleted the unique miR-29 and miR-124 binding sites using restriction digestion and re-ligation. Luciferase activities were then measured in HeLa cells transfected with the modified and unmodified constructs. As shown in Figure 6C, the deletion of the miR-29 binding site significantly increased luciferase activity compared with the miR-29 mimic in HeLa cells. Similarly, luciferase activity was significantly higher in HeLa cells transfected with miR-124 binding site deletion construct compared with the luciferase activity in HeLa cells transfected with miR-124 mimic (Figure 6C). To further confirm the effects of those miRNAs on endogenous WDFY1 expression, we treated mesangial cells with the miR-29 and miR-124 mimics and then measured WDFY1 expression in the cell lysate by Western blot. As shown in Figure 6D, decreased WDFY1 expression was observed in mesangial cells treated with miR-29 or miR-124 mimics compared with WDFY1 levels in the untreated mesangial cells.

## 4. Discussion

The functions of WDFY1 have now been identified. It mainly serves as a regulator of endocytic membrane trafficking and as an adaptor protein for TLR3 and TLR4 signaling [3,6]. We recently reported a potential requirement of WDFY1 for FDC antigen processing and presentation in mice with inducible lupus [15]. Despite the recent progress in functional studies, the mechanisms of WDFY1 expression and regulation remain to be determined. In our present work, we systematically characterized the promoter, 5′-UTR, and 3′-UTR of this highly conserved gene in the mouse and validated TFs and miRNAs that regulated WDFY1 expression in mesangial cells and in the HeLa cell line for the first time. We focused our initial studies on delineating the minimally required basal WDFY1 promoter driving endogenous gene expression. We found that all of the known Sp1 binding sites but none of the TCF7 binding sites resided within the minimally required basal promoter region, i.e., between −1106 and +1. The important roles for four TFs and two miRNAs in Wdfy1 expression were confirmed by either overexpression or siRNA knockdown of the TFs and miRNAs.

The miR-29-3p binding site was the only one predicted with high affinity. MiR-29 controls innate and adaptive immune responses by targeting the interferon pathway [26]. Downregulated miR-29 is also known to promote B cell overactivation in systemic lupus erythematosus (SLE) [27]. This miRNA has also been demonstrated to inhibit widespread gene expression by targeting Sp1 [28]. Meanwhile, other studies suggest that IL-6 can downregulate miR-29 expression in different cell types [29]. Therefore, the significantly enhanced WDFY1 expression in IL-6-treated primary mesangial cells (Figure 6) may be due to a combined synergistic effect of directly downregulating miR-29 and indirectly enhancing Sp1, which is a main TF contributing to WDFY1 upregulation. These observations, taken together, make miR-29 a fruitful therapeutic candidate for treating inflammatory diseases, including SLE. With respect to the other miRNAs we studied, miR-124 polymorphism is associated with SLE susceptibility [24]. Its expression is significantly reduced in patients with active lupus nephritis, and it is negatively correlated with serum inflammatory cytokine expression [22,23]. MiR-124 expression is also abnormally low in the kidneys of lupus mice [30]. We demonstrated here that miR-124 inhibited WDFY1 expression in mesangial cells, consistent with the report that miR-124 played an inhibitory role in renal mesangial growth and inflammation, making miR-124 an attractive diagnostic marker in lupus nephritis.

Short Interspersed Nuclear Element (SINE) retrotransposons constitute one of the main components of the genomic repetitive fraction, involved in the maintenance of genome stability and proper functionality [31]. Retrotransposons regulate gene expression by affecting gene transcription, pre-mRNA processing, or mRNA metabolism [32]. B1-SINE retrotransposon is widely represented in the mouse genome. In addition to the 3′UTR B1-SINE element, we also identified a reverse B1-SINE sequence in the body of the *wdfy1* gene. WDFY1 transcript variants containing this second SINE element have also been reported [33]. It is possible that the two B1-SINE sense and antisense pair together to coordinately regulate the expression of *wdfy1* transcript variants. In addition, short upstream ORFs (uORFs) were identified in the beginning of the WDFY1 ORF, which may impact WDFY1 expression. The translation of those uORFs may inhibit ribosomal scanning to facilitate canonical ORF translation. Such a mechanism was demonstrated by Wu et al. [34].

WDFY1 expression regulation in the mouse kidney is certainly more complex than we have demonstrated in this study. Gene regulation involves multiple cis and trans mechanisms in addition to spatial controls [35]. The four-fold enhanced luciferase activity we detected here is probably due to a spatial assembly of different elements. In the ~3.5 kb promoter and 5′-UTR regions of the mouse wdfy1 gene studied here, there are hundreds of TF binding sites potentially orchestrating WDFY1 expression. Similarly, many more miRNA binding sites were retrieved through the online prediction, albeit with less conservation through other mammalian species. It is likely that WDFY1 expression is regulated by multiple TF and miRNA complexes that involve additional adaptor proteins, including those affecting chromosome architecture. However, under current experimental and financial situations, it is not practical to test all these components. More work is needed to fully elucidate WDFY1 transcriptional and translational regulation and how post-translational modification fine-tunes gene expression.

## 5. Conclusions

Taken together, this is the first study to characterize the mouse *wdfy1* gene promoter, 5′-UTR, and 3′-UTR. We identified four essential TFs and two siRNAs as major elements of *wdfy1* gene expression and regulation in mouse renal mesangial cells. Further study is needed to elucidate the functions of these elements and other TFs in the inducible expression of *wdfy1*.

## Data Availability

No new datasets were created that are posted.

## Supplementary Materials

The following supporting information can be downloaded at https://www.mdpi.com/article/10.3390/cells14110798/s1: Table S1: Wdfy1 promoter and 5′-UTR TF binding sites and construct.
