# Supplementary material for: Regulation of WDFY1 Expression by miRNAs, Transcription Factors, and IL-6 in Murine Mesangial Cells
Source: Cells. 2025 May 29;14(11):798. doi: 10.3390/cells14110798 (PMC12153811; doi:10.3390/cells14110798)
Supplement: Supplementary file 1 [file cells-14-00798-s001.zip › cells-3621205-supplementary.pdf]

Table S1. Wdfy1 promoter and 5′-UTR TF binding sites and construct

**Insert Size**	TF sites
3.5 kb	Full-size insert.
2.9 kb	Eliminates 1 TCF7 site, 2 AP1 sites and 5 Hes1 sites.
2.4 kb	Eliminates 4 Hes1 sites and 6 AP1 sites.
1.9 kb	Eliminates 1 TCF7 site, 4 Hes1 sites, and 6 AP1 sites,.
1.4 kb	Eliminates 3 Hes1 sites, 3 TCF sites, and 4 AP1 sites
1.1 kb	Eliminates 1 Hes1 site, 2 AP1 sites, and 2 TCF7 sites.
0.9 kb	Eliminates 2 Hes1 sites.
0.6 kb	Eliminates 1 Sp1 site, 1 Hes1 site, 1 AP1 site, and 2 TCF7 sites.
0.39 kb	Eliminates 1 AP1 site, 2 Hes1 sites.
0.24 kb	Eliminates 1 Hes1 site.
0.14 kb	Eliminates 1 AP1 site
0.09 kb	Retains 1 AP1 site, 1 CTCF site, and 2 Sp1 sites. It also retains the TSS1 and the downstream protein start codons.
34 bp	Empty "Basic" vector.
